# Development of a Fast, Low-Cost, and Green Method to Quantify Allura Red AC Dye in Candies through Digital Images Using a Smartphone

**DOI:** 10.1021/acsomega.5c06279

**Published:** 2025-10-09

**Authors:** Maria Eduarda B. Coutinho, Bruna R. de S. Gomes, Jandyson M. Santos

**Affiliations:** Department of Chemistry, 67744 Federal Rural University of Pernambuco, Recife, Pernambuco 52171-900, Brazil

## Abstract

The artificial azo dye Allura Red AC (E129), widely used in candy, may pose health risks, such as hypersensitivity and hyperactivity, underscoring the need for its monitoring and quantification to ensure food safety. This study adapted a traditional sample preparation approach aligned with the principles of green analytical chemistry (GAC) and introduced a low-cost, smartphone-based colorimetric method employing digital image acquisition. The technique enables the simultaneous capture of both the analytical curves and the samples in a single image by using a 3D-printed digital imaging chamber. The data were processed using the RGB color model and analyzed by partial least-squares with REDGIM software. For the adapted UV–vis method, accuracy at two concentration levels ranged from 77.33% ± 1.53 to 98.35% ± 0.07, with a limit of quantification (LQ) of 2.26 × 10^–6^ mg mL^–1^ and a limit of detection (LD) of 7.47 × 10^–7^ mg mL^–1^. For the DIA-RD method, accuracy ranged from 78.04% ± 1.42 to 98.42% ± 0.06, with a LQ of 1.67 × 10^–4^ mg mL^–1^ and a LD of 5.51 × 10^–5^ mg mL^–1^. E129 concentrations in ten candy samples ranged from 7.00 × 10^–4^ to 5.16 × 10^–3^ mg mL^–1^ using the UV–vis method and from 7.33 × 10^–4^ to 4.83 × 10^–3^ mg mL^–1^ using the DIA-RD method, all below the legal limit of 3.0 × 10^–1^ mg mL^–1^ established by Brazilian and international regulations. The DIA-RD method significantly reduced sample preparation time, enabling data acquisition and processing in 5 min compared to 6 h with the UV–vis method. It complies with GAC principles and shows a strong potential for routine quality control of candy products.

## Graphical abstract

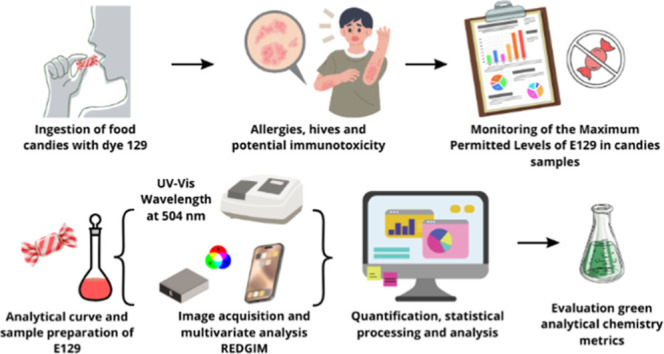

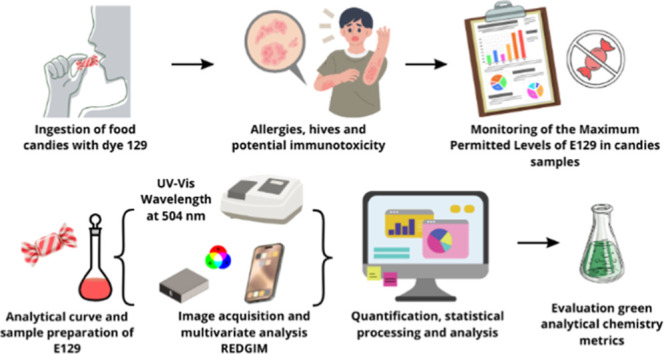

## 1 Introduction

Food additives are substances incorporated into food products whose main function is to improve the physicochemical aspects of food, such as consistency, aroma, and texture.^1^ Food colorants (FCs) attribute color to the final product but may be linked to potential adverse risks, including hypersensitivity reactions, hyperactivity in children, and inflammatory bowel diseases.^2,3^

The main FCs are artificial dyes such as amaranth, sunset yellow FCF, tartrazine, brilliant blue FCF, ponceau 4R, erythrosine, and Allura Red AC (C_18_H_14_N_2_Na_2_O_8_S_2_).^4^ This last dye confers a red/pink color in food/beverages widely used in the food industry.^5^ Nevertheless, there are several dangers associated with its excessive consumption, such as allergies and potential immunotoxicity.^6^

As recommended by ANVISA—RDC N^o^ 778/2023, as well as by the guidelines established in the Codex General Standard for Food Additives,^7,8^ the maximum allowable limit for E129 in candies is 3.0 × 10^–1^ mg mL^–1^. In addition, the acceptable daily intake for this additive has been set by the Joint FAO/WHO Expert Committee on Food Additives (JECFA) at 7.0 mg kg^–1^ of body weight, a fundamental parameter for toxicological evaluation and safety of human consumption.^9^

In addition to this official method, several studies have proposed alternative analytical approaches, such as high-performance liquid chromatography (HPLC),^10^ liquid chromatography coupled to quadrupole-Orbitrap high resolution mass spectrometry (LC-Q-Orbitrap MS),^11^ the employment of nanomaterial-based electrochemical sensors,^12^ as well as absorption spectrophotometry using hybrid poly (vinyl alcohol) nanofilms and silver nanoparticles (PVA-Ag).^13^ Although efficient, these methods are associated with sample pretreatment protocols that do not meet the green analytical chemistry (GAC) criteria, involving the use of toxic organic solvents, time-consuming sample extraction processes, high energy costs, and expensive equipment.^14^

On the other hand, the main advantages of the digital image acquisition (DIA) methods using smartphones are related to high dynamicity, cost-effectiveness, reduced use of solvents, and accuracy, making it a greener alternative compared to conventional methods. The protocols try to be aligned with the principles of GAC, whose adherence can be assessed using the Green Analytical Procedure Index (GAPI)^15^ and the Analytical Greenness Calculator tool by AGREE software.^16^ Methods using smartphones as an analytical tool have gained increasing visibility in fields such as clinical analysis,^17^ pharmaceutical analysis,^18^ environmental analysis,^19^ fuel analysis,^20^ forensic analysis,^21^ and food/beverage analysis.^22,23^

The DIA methods use the Red (R), Green (G), and Blue (B) channels to capture and process colorimetric information, and the analytical signal can be expressed in terms of reflectance rather than absorbance, as occurs in molecular absorption spectrophotometry.^24^ The analytical responses of these channels range from 0 to 255, with their combinations attaining different colors.^25^ A prior colorimetric reaction is necessary due to the selective absorption of wavelengths in the visible region of the electromagnetic spectrum, which produces a characteristic color, which is a reflectance captured by the imaging device and quantitatively analyzed.^26^ The performance of a DIA method depends not only on advanced devices but also on better definitions of the lighting, focus, and standardized camera-to-sample distance to ensure high repeatability and reproducibility.^27^ The use of digital imaging chambers, which can be made of wood^28^ or produced by 3D printing,^22,29^ is strongly recommended to control brightness, minimize shadows, and enable reliable in situ analysis.^29–31^

Furthermore, DIA-based analysis requires specialized software for intelligent data processing. One example is REDGIM,^32^ which allows obtaining the analytical response from a single image, correlating color intensity with analyte concentration through multivariate image analysis using partial least-squares (PLS). It employs key statistical metrics, such as the root mean square error of prediction (RMSEP), root mean square error of calibration (RMSEC), and the coefficient of determination (*R* ^2^), thereby ensuring greater accuracy and reliability in analyte quantification.

Considering the need for environmentally friendly, more accessible analytical methods and the importance of controlling the quality of dyes in foods, we have developed an adaptation in a UV–vis reference method for the quantification of E129 in commercial candies and established a smartphone-based digital images method. The methods were evaluated by their alignment with the principles of GAC using the GAPI approach and AGREE software. Herein, the DIA method developed using REDGIM software was named DIA-RD.

## 2 Materials and Methods

### 2.1 Reagents and Solutions

The 5% (*v*/*v*) ammonia methanol solution was prepared by adding methanol (Neon, São Paulo, Brazil) to ammonium hydroxide (Quimica Moderna, São Paulo, Brazil). The stock solutions of the standard were made by dissolving the E129 dye (80%) (Sigma-Aldrich, Saint Louis, EUA) in different solvents: 5% (*v*/*v*) ammonia, methanol, and ultrapure water (resistivity > 18.0 MΩ cm). E102 (85%) (Sigma-Aldrich, Saint Louis, EUA) and E133 (Sigma-Aldrich, São Paulo, Brazil) were used as possible interferents in the validation of the studies.

### 2.2 Samples

Ten samples of different brands of soft and gelatinous candies, with flavors of strawberry, strawberry yogurt, raspberry, cherry, and blackberry, containing only the dye E129 described on the label, without the presence of other dyes in their composition, were acquired in commercial markets of Recife city, Pernambuco state, Brazil, and were coded from A1 to A10.

### 2.3 Analytical Curves

The dye E129 (80% purity) was dissolved at 10.0 mg mL^–1^ by dissolving 0.625 g in 50.0 mL of two different media: 5% (*v*/*v*) ammonia, methanol extraction, and ultrapure water. From these stock solutions, working solutions at a concentration of 1.00 × 10^–1^ mg mL^–1^ were prepared by appropriate dilution. Analytical curves were then constructed using six concentration levels: 2.00 × 10^–4^, 5.00 × 10^–4^, 1.00 × 10^–3^, 5.00 × 10^–3^, 1.00 × 10^–2^, and 2.00 × 10^–2^ mg mL^–1^.

### 2.4 Analytical Workflows

The methods used in this study were organized into two analytical protocols instead of ultrapure water as the solvent of preparation (Figure 1): (a) adapted UV–vis method from the reference protocol^33^ and analysis by molecular absorption spectrophotometry in the UV–vis region; and (b) DIA-RD method with smartphone and multivariate analysis using REDGIM software. The sections below describe the methodological information for both protocols.

**Figure 1:**
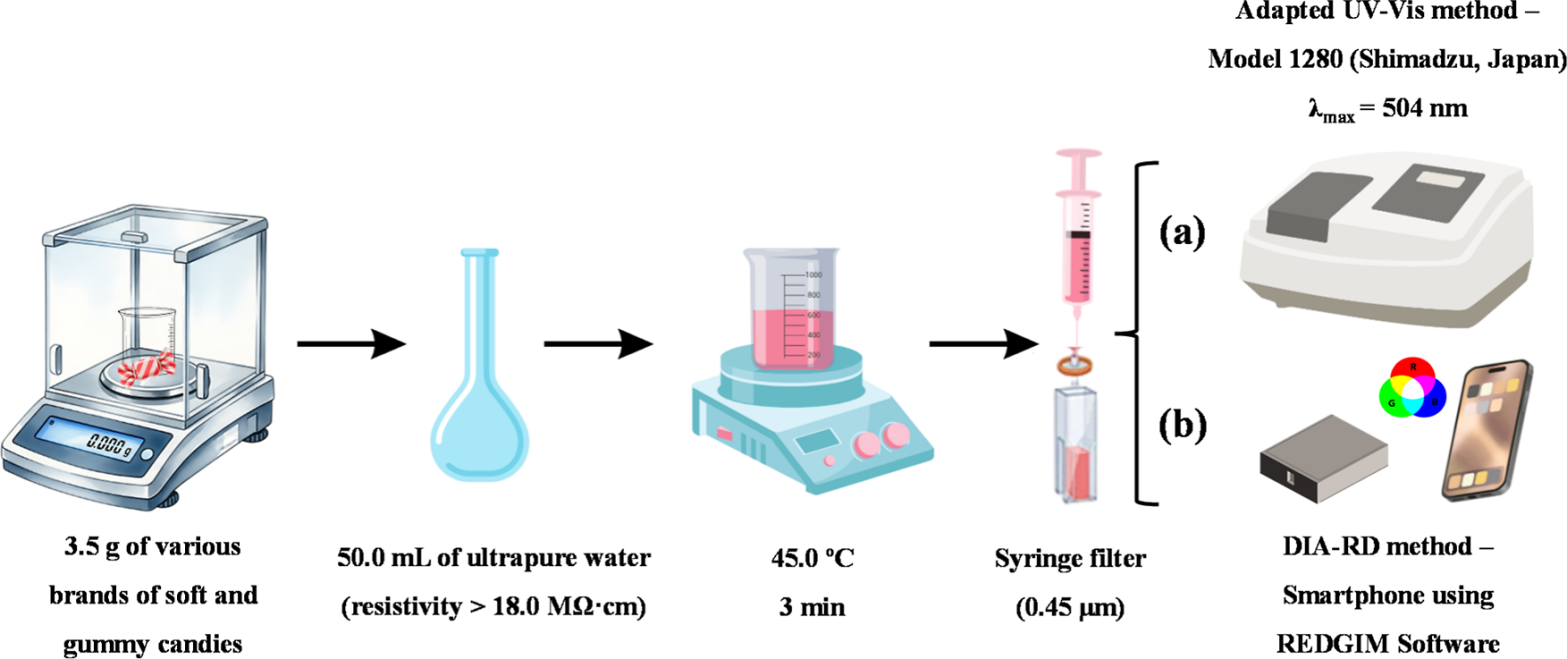
Scheme of the analytical workflows for adapted UV–vis (a) and DIA-RD (b) methods. Created in https://www.canva.com.

#### 2.4.1 Adapted UV–Vis Method

An adaptation of the sample preparation method described in the reference protocol,^33^ originally based on the use of 5% (*v*/*v*) ammonia methanol as a solvent and filtration with filter paper, with the solvents replaced by water (Figure 1a), was carried out. Due to this modification, three different types of filtrations were tested: 80 g of qualitative filter paper (Whatman, China), 0.45 μm syringe filter (KASVI, China), and 2.7 μm glass microfiber filter (Whatman, China).

The results obtained for the three types of filtrations were compared with those obtained by the reference method, which used filter paper in a 5% (*v*/*v*) ammonia methanol solution. This method was also adapted for application in the evaluated systems, including a 0.45 μm syringe filter and a 2.7 μm glass microfiber filter in water. The analyses were performed by spectrophotometry in the UV–vis region using a wavelength of 504 nm, corresponding to the maximum absorption of this dye.

#### 2.4.2 DIA-RD Method

To perform the acquisition of digital images, a smartphone was used in conjunction with a low-cost digital imaging chamber constructed using a 3D printer, which has space for nine cuvettes, allowing simultaneous analysis.^34^ We adapted the image acquisition conditions to evaluate the distance between the smartphone camera and the samples (between 5 and 16 cm), and the intensity of internal LED strips (minimum or maximum ≈1124 l×) by applying a Design of Experiments (DoE) using a 2^2^ full factorial design with five central points using Design Expert-7 software, trial version.^35^ The analytical response evaluated was the relative error (%) between the predicted and actual values, typically expressed as a percentage, obtained from analysis of the analytical curve solutions of the E129 dye standard in ultrapure water.

After the optimal image acquisition conditions were determined, the solutions prepared as described in Section 2.4.1, using the defined best filtration option, were captured with a smartphone equipped with a 50-megapixel camera and *f*/1.8 aperture (Figure 1b), establishing the DIA-RD method. A single smartphone, equipped with cameras with apertures between *f*/1.7 and *f*/1.9 as cited,^36^ was used, providing comparable analytical performance. The multiple capture mode was used, which allowed the selection of the region of interest for the six points of the analytical curve, the analytical blank (ultrapure water), and real samples of commercial candies containing the E129 dye. ROIs were selected in dimensions as squares with a 45-pixel edge. From a single image, the color information extracted from these regions was converted into histograms based on the R, G, or B components. Then, the software calculated the reflectance values and provided the statistical parameters RMSEP, RMSEC, bias, relative error (%), and *R* ^2^.

### 2.5 Analytical Statistics

For statistical analysis, the RS Team software environment (version 2022.12.0)^37^ was used. Linear models were applied to evaluate the relationship between concentration and analytical response of the methods: adapted UV–vis and DIA-RD. Statistical significance was verified by analysis of variance (ANOVA), homoscedasticity was assessed by the Breusch–Pagan test, and normality of residuals was assessed by the Shapiro–Wilk test.

The precision and accuracy of the methods were also estimated, with variances compared with the *F* test. The DoE 2^2^ conditions were evaluated by means of normal probability graphs, Pareto charts, and response surfaces, aiming to identify the best conditions for using the digital imaging chamber in the DIA-RD method.

### 2.6 Analytical Validation

The validation criteria were defined according to the standards provided by the analytical guidelines of the Brazilian National Standards Association.^38^ The analytical metrics were evaluated as detailed below.

- Recovery (%): This was evaluated by adding a known amount of analyte to the sample. The addition of the standard was performed at two levels: low and high, with additions of 5.00 × 10^–4^ and 1.00 × 10^–2^ mg mL^–1^, respectively. The recovery was calculated considering:

$$R=\frac{C_{1}-C_{2}}{C_{3}}\times 100\%$$

where *C*_1_ is the concentration measured in the fortified sample; *C*_2_ is the concentration of the unfortified sample; and *C*_3_ is the added concentration. The accepted value is ≥80% or ≤120%.

- Precision: this was calculated using the coefficient of variation (CV), calculated as CV (%) = (SD/*x̅*) × 100%, where *x̅* is the sample group mean, and SD is the standard deviation; acceptance value: ≥20%;
- LD and LQ: these were calculated using the equations 3.3 × σ/*m* and 10 × σ/*m*, respectively, where σ is the standard deviation of ten measurements of analytical blanks or the standard deviation from the point of lowest value of the analytical curve, and m is the slope of the analytical curve (angular coefficient).

### 2.7 Green Analytical Chemistry Metrics

The GAC assessment was carried out based on the GAPI, analyzing principles such as collection, preservation, transportation, storage, type of method, sample preparation, solvents/reagents used, additional treatments, amount, health hazard, safety risk, energy, occupational risk, waste, and its treatments. In addition, the metric system of the free software AGREE was also evaluated, which indicates the environmentally friendly method with overall scores close to one by converting the 12 GAC principles into numbers, with scores close to one corresponding to the greenest method. Both parameters (GAPI and AGREE) were represented by the colors: white (the metric does not apply), green (environmentally friendly method), yellow (intermediate), and red (nongreen method).

## 3 Results and Discussion

### 3.1 Adapted UV–Vis Method from the Reference

First, we have performed a comparison of the different types of filtrations (80 g qualitative filter paper, 0.45 μm syringe filter, and 2.7 μm glass microfiber filter) using 5% (*v*/*v*) ammonia methanol extraction (reference method^32^), and also ultrapure water for the proposed method. In both, the highest absorbance values were obtained by using the syringe filter (Table 1). The proposed UV–vis method presented higher absorbance values than the reference method, demonstrating greater efficiency in solubilizing the E129 dye. Furthermore, the use of water as a solvent, combined with syringe filter filtration, represents a more viable and environmentally friendly alternative, reducing the use of organic solvents and minimizing adsorption losses during the filtration step.

**Table 1:** UV–Vis Absorbances of the Experimental Filtration Steps for Reference and Adapted UV–Vis Methods Based on the Mean of the Values and Standard Deviations in the Quantification of E129 in a Random Sample of Candies (95% Confidence Level, *n* = 3)

types of filtrations	absorbance—reference method	absorbance—adapted UV–vis method
filter paper	8.0 × 10^–2^ ± 5.0 × 10^–4^	-^a^
glass microfiber filters	9.8 × 10^–2^ ± 1.0 × 10^–2^	7.4 × 10^–2^ ± 2.1 × 10^–2^
syringe filter	1.1 × 10^–1^ ± 5.0 × 10^–4^	2.0 × 10^–1^ ± 4.0 × 10^–3^

a -: Unable to quantify due to the turbidity of the solution.

During the preparation of the analytical curve solutions, an increase in the intensity of the pink/red coloration was observed, corresponding to rising concentrations of the E129 dye. This trend indicates a proportional relationship between dye concentration and color intensity, thereby facilitating quantitative analysis using both the adapted UV–vis and DIA-RD methods, each performed in triplicate (Figure 2A). The analytical curve of E129 using water as a solvent was prepared in the concentration range of 2.00 × 10^–4^ to 2.00 × 10^–2^ mg mL^–1^ and showed *R* ^2^ values greater than 0.99 (Figure 2B), indicating satisfactory linearity. For comparison, an additional analytical curve was constructed using 5% (*v*/*v*) ammonia methanol, according to the reference method (Figure S1).

**Figure 2:**
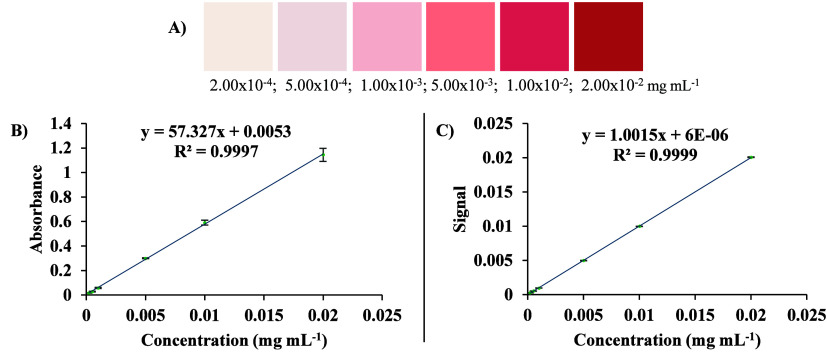
Analytical curves for the E129 standard solutions of the methods: color scale according to concentration for the E129 (A) adapted UV–vis (B); DIA-RD (C). Error bars are not visible for some data points of the analytical curve, due to minimal standard deviations between triplicates.

As a result, statistical analyses validated the linear model of the adapted UV–vis method (ANOVA, *p* = 1.20 × 10^–6^), demonstrating homoscedasticity (Breusch–Pagan test, *p* = 0.44) and normality of the residuals (Shapiro–Wilk test, *p* = 0.47), indicating that the residuals followed a normal distribution. These results demonstrate the high accuracy of the model, with minimal residuals and a good fit to the experimental data, confirming the robustness and reliability of the analytical curve.

The LQ and LD of the adapted UV–vis method were 2.26 × 10^–6^ mg mL^–1^ and 7.47 × 10^–7^ mg mL^–1^, respectively, with a coefficient variation (% CV) of 1.38 × 10^–5^. As shown in Table 2, the adapted UV–vis method was accurate at both evaluated concentration levels: low (5.0 × 10^–4^ mg mL^–1^) and high (1.0 × 10^–2^ mg mL^–1^). Furthermore, the recovery value ranged from 77.33% ± 1.53 to 98.35% ± 0.07, within acceptable limits for analytical methods. These results demonstrate the precision of the adapted UV–vis method for quantifying the analyte.

**Table 2:** Recovery Values for Low (5.00 × 10^–4^ mg mL^–1^) and High (1.00 × 10^–2^ mg mL^–1^) Levels for Adapted UV–Vis Method (A) and DIA-RD Method (B)^a^

samples	levels	recovery (%) adapted UV–vis	recovery (%) DIA-RD
A1	5.00 × 10^–4^	78.93 ± 1.96	79.48 ± 1.94
	1.00 × 10^–2^	98.35 ± 0.07	98.33 ± 0.03
A2	5.00 × 10^–4^	78.51 ± 0.79	78.62 ± 2.12
	1.00 × 10^–2^	98.19 ± 0.26	98.35 ± 0.02
A3	5.00 × 10^–4^	77.33 ± 1.53	78.04 ± 1.42
	1.00 × 10^–2^	98.32 ± 0.01	98.42 ± 0.06

a 95% confidence level, *n* = 3.

### 3.2 DIA-RD Method

The optimal conditions for image acquisition in the DIA-RD method, as determined by experimental design (DoE) (Table S1), were evaluated using Normal and Pareto charts (Figure S2). The results indicated a significant effect of distance (factor A), with a negative influence, suggesting an improved performance at a distance of 10 cm. This condition yielded a desirability score of 92.80% (Figure S3). Although the lighting factor did not show a statistically significant effect, the LED was set to the maximum intensity to enhance the digital imaging chamber internal illumination. Under these conditions, the analytical curve exhibited a coefficient of determination (*R* ^2^) of 0.999, demonstrating excellent linearity of the DIA-RD method (Figure 2C). The higher sensitivity of the UV–vis spectrophotometer is due to its use of monochromatic light, high spectral resolution, and precise wavelength selection, minimizing background noise. In contrast, DIA relies on broadband light and RGB channels, reducing the spectral selectivity and increasing the effects from ambient light, scattering, and pixel quantization.

Thereafter, processing the images using the DIA-RD method, the multivariate chemometric model generated by multiple capture was evaluated using the DIA-RD. The metrics associated with the prediction of the analytical curve indicated that the PLS model parameters performed satisfactorily (Table S2), according to the evaluated criteria (linear range, curve equation, *R* ^2^, RMSEP, RMSEC, and RE %), resulting in the selection of three latent variables. Statistical analyses validated the linear model of the DIA-RD method (ANOVA, *p* = 4.85 × 10^–10^), demonstrating homoscedasticity (Breusch–Pagan, *p* = 0.18) and normality (Shapiro–Wilk test, *p* = 0.29), indicating that the residuals followed a normal distribution.

The validation parameters for the developed DIA-RD method showed LQ and LD of 1.67 × 10^–4^ mg mL^–1^ and 5.51 × 10^–5^ mg mL^–1^, respectively, with a % CV of 1.67 × 10^–5^. In addition, recovery tests also showed values within acceptable limits at two concentration levels: low (5.00 × 10^–4^ mg mL^–1^) and high (1.00 × 10^–2^ mg mL^–1^), as shown in Table 2. As a result, the recovery values obtained at both low and high concentrations indicated that the DIA-RD method demonstrated satisfactory accuracy, with recoveries within the acceptable range of 78.04% ± 1.42% to 98.42% ± 0.06%.

Table S3 provides a detailed comparison between the proposed DIA-RD method and other DIA approaches available in the literature. The analytical parameters of the developed DIA-RD method demonstrate comparable performance to other studies that have also employed this approach.^39^ Previous work using DIA for the quantification of artificial dyes tartrazine and Allura Red AC in food products (liquid sweetener, liqueur, popsicles, and liquid candy) reported a LD and LQ of 2.0 × 10^–4^ and 6.8 × 10^–4^ mg mL^–1^, respectively,^40^ while another study achieved an LD of 6.0 × 10^–4^ mg mL^–1^. The comparisons in Table S3 highlight the satisfactory performance of the proposed method, which combines operational simplicity, reduced cost, and the possibility for in situ analysis.

In addition, when compared with established instrumental techniques, such as HPLC, similar analytical sensitivities were observed. For example, LD between 5.0 × 10^–5^ and 1.5 × 10^–4^ mg mL^–1^, and LQ between 1.0 × 10^–4^ and 1.5 × 10^–4^ mg mL^–1^ were reported for the determination of six synthetic dyes (tartrazine, sunset yellow, amaranth, indigotine, brilliant blue FCF, and Allura Red AC) in various food matrices (flavored alcoholic and nonalcoholic beverages such as soda, energy drinks, and juices, as well as gelatin and different types of candy) by HPLC-UV-DAD.^41^ Additionally, a dispersive solid-phase extraction method coupled with HPLC-UV–vis reported an LD of 1.5 × 10^–4^ mg mL^–1^ and LQ of 5.0 × 10^–4^ mg mL^–1^ for the determination of synthetic dyes (tartrazine, sunset yellow, amaranth, ponceau 4R, indigo carmine, and brilliant blue FCF) in sports drinks with flavors (tangerine, orange, grape, green grape, passion fruit, and mixed fruit).^42^

### 3.3 Sample Analysis and Statistical Parameters

Table 3 presents the results for the quantification of E129 in ten commercial candy samples and their statistical parameters obtained using the adapted UV–vis method in comparison with the DIA-RD method.

**Table 3:** Quantification and Statistical Parameters for Analysis of E129 Dye in Ten Candy Samples by the Adapted UV–Vis and DIA-RD Methods^a^

samples	adapted UV–vis method (mg mL^–1^)	DIA-RD method (mg mL^–1^)	*F*-value	*|p*-value*|* (*F*-test)	|*t* value|	*p*-value (*t*-test)	relative error (%)
A1	3.20 × 10^–3^ ± 1.00 × 10^–4^	3.36 × 10^–3^ ± 1.52 × 10^–4^	0.42	0.60	2.50	0.13	5.20
A2	5.16 × 10^–3^ ± 4.16 × 10^–4^	4.83 × 10^–3^ ± 4.93 × 10^–4^	0.71	0.83	1.79	0.21	6.45
A3	1.33 × 10^–3^ ± 2.00 × 10^–3^	1.26 × 10^–3^ ± 1.52 × 10^–4^	1.85	0.70	1.00	0.42	5.00
A4	8.00 × 10^–4^ ± 1.00 × 10^–4^	7.66 × 10^–4^ ± 1.52 × 10^–4^	0.43	0.60	0.50	0.66	4.16
A5	1.46 × 10^–3^ ± 2.08 × 10^–4^	1.56 × 10^–3^ ± 1.00 × 10^–4^	1.85	0.70	0.86	0.47	6.81
A6	5.00 × 10^–4^ ± 1.00 × 10^–4^	5.00 × 10^–4^ ± 1.00 × 10^–4^	1.00	1.00	0.00	1.00	0.00
A7	1.30 × 10^–3^ ± 1.00 × 10^–4^	1.20 × 10^–3^ ± 1.00 × 10^–4^	1.00	1.00	0.86	0.47	7.69
A8	7.00 × 10^–4^ ± 1.00 × 10^–4^	7.33 × 10^–4^ ± 1.52 × 10^–4^	0.42	0.60	0.25	0.82	4.76
A9	4.46 × 10^–3^ ± 3.05 × 10^–4^	4.56 × 10^–3^ ± 6.02 × 10^–4^	0.25	0.40	0.57	0.62	2.23
A10	2.50 × 10^–3^ ± 1.00 × 10^–4^	2.36 × 10^–3^ ± 2.08 × 10^–4^	0.23	0.37	2.00	0.18	5.33

a Values with 95% confidence level, *n* = 3.

Therefore, for all samples, the relative errors between the results obtained by the methods were low, with a maximum value of up to 7.69%. Furthermore, the t-value obtained utilizing the bilateral paired *t*-test (95% confidence level, *p* > 0.05) was lower than the critical *t*-value (4.31). As well, the *F* values were lower than the critical *F*-value (19.00), which demonstrated that no statistically significant differences were found between the methods.

In conclusion, the concentrations of the E129 dye in the ten candy samples ranged from 7.00 × 10^–4^ to 5.16 × 10^–3^ mg mL^–1^ for the adapted UV–vis method, and from 7.33 × 10^–4^ to 4.83 × 10^–3^ mg mL^–1^ for the DIA-RD. When we compare with the limits of Brazilian and International legislations, all values were below the maximum allowable limit established by ANVISA, FAO, and WHO, which means lower than 3.00 × 10^–1^ mg mL^–1^.

### 3.4 Assessment of Greenness Metrics

The evaluations of the GAC metrics on real samples obtained by the methods Reference, adapted UV–vis, and DIA-RD, are presented in Table S4, while the GAPI evaluation pictograms corresponding to the three methods are shown in Figure 3.

**Figure 3:**
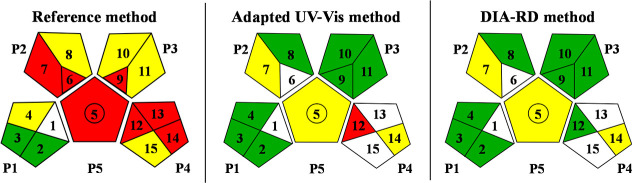
GAPI evaluation of the methods: reference, adapted UV–vis, and DIA-RD, for determination of the E129 dye in candies. Categories: P1: sample handling, P2: sample preparation, P3: solvents/reagents, P4: instrumentation, and P5: type of general method.

In P1, the proposed methods do not require specific preservation, transport, and storage of the sample, resulting in a green classification in parameters 2, 3, and 4. In the reference method, it is necessary to store the samples at room temperature and protect them from light, which justifies the yellow color in parameter 4. For parameter 1, related to collection, the metric does not apply in the three methods, being represented in white.

In the P2 evaluation, it was observed that, in parameters 6 and 7, the red coloration attributed to the reference method contrasts with the white coloration observed in parameter 6 of the proposed methods (adapted UV–vis and DIA-RD). This discrepancy is due to the absence of the extraction step in the developed methods, making the corresponding metric inapplicable. In addition, these methods replace toxic solvents with more environmentally friendly alternatives, which is reflected in the yellow coloration of parameter 7.

In P3, parameter 9, the reference method was classified as red due to the consumption of more than 100 mL of solvents and the high generation of waste associated with the use of a 5% (*v*/*v*) ammonia methanol solution as an extractant. In contrast, the proposed methods used volumes lower than 10 mL and presented a low generation of waste, which justified the assignment of a green color.

In parameters 10 and 11, the reference method was classified as moderately toxic and presented greater flammability or instability according to the NFPA, resulting in yellow. The proposed methods, in turn, did not present significant risks to health or safety, as they were assigned the color green for these parameters.

As referred to in P4, parameter 12 was classified as red for the UV–vis methods due to high energy consumption and the fact that only one analysis can be performed at a time. In contrast, the DIA-RD method, which uses a smartphone, received a green classification, as it consumes less energy and allows multiple analyses to be performed simultaneously. In parameter 13, the reference method presented an occupational risk with vapor emission, classified as red. This metric does not apply to the proposed methods, which is why it was assigned a white color. In parameter 14, the waste volume of the reference method was greater than 10 mL (red), while the proposed methods generated between 1 and 10 mL (yellow).

In another case, for parameter 15 related to waste treatment, the reference method requires a passivation step, although without clear guidelines, for which it was given a yellow coloration. In the proposed methods, this step is nonexistent, which justifies the white coloration observed. Finally, picogram 5 (P5) indicated circles for all methods, signaling that they were quantitative procedures.

Finally, the AGREE evaluation (Figure 4 and Table S5) was applied to the three methods (reference, adapted UV–vis, and DIA-RD). The reference method obtained a score of 0.37, with a predominance of red coloration, evidencing low alignment with the principles of GAC. In contrast, the proposed methods were greener, with scores of 0.77 for the adapted UV–vis method and 0.81 for DIA-RD, due to the use of a more environmentally friendly solvent and the avoidance of the generation of toxic waste.

**Figure 4:**
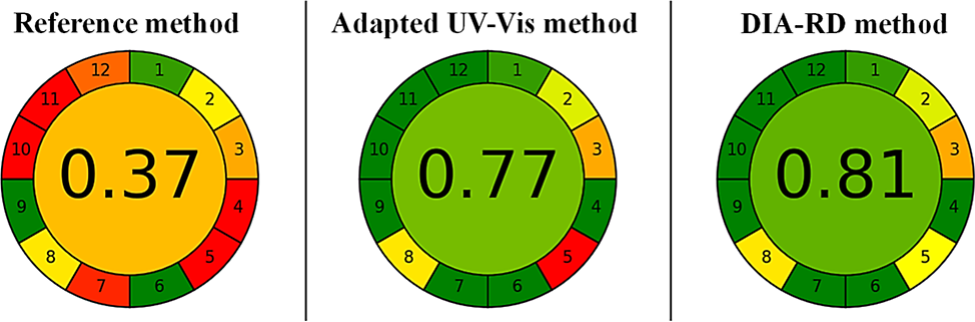
Metric evaluation using AGREE of the methods: reference, adapted UV–vis, and DIA-RD, for determination of the E129 dye in candies. (Principles: 1—pretreatment, 2: sample quantity, 3: analysis, 4: sample preparation steps, 5: automation/miniaturization, 6: derivatization, 7: residues, 8: yield, 9: energy, 10: reagents, 11: toxicity, and 12: safety.)

Among these, the DIA-RD method stands out, which, in addition to these characteristics, required a shorter analysis time compared to the reference method, from 5 min to 6 h, giving it additional advantages in terms of analytical efficiency. In short, these factors contributed to the development of safer and more automated instrumentation, enabling the replacement of traditional analytical techniques in exchange for smartphone-based solutions combined with the use of more environmentally friendly solvents.

## 4 Conclusion

We have adapted the sample preparation of a reference method, replacing toxic residues and reducing the analysis time, aiming to quantify E129 dye in candies by UV–vis analysis. In addition, we developed a DIA-RD method using a smartphone camera for the same purpose. These proved to be equivalent in analytical performance, with the DIA-RD method standing out for being more environmentally friendly, reducing the analysis, minimizing energy consumption, and providing adequate operational safety. In addition, it replaced expensive equipment, such as a UV–vis spectrophotometer, whose use is often associated with less green pretreatment protocols. The developed approach was successfully applied to real candy samples using PLS for predictive modeling with variables extracted from the RGB channels. For both methods, the concentrations of the E129 dye in the ten candy samples ranged from 7.00 × 10^–4^ to 5.16 × 10^–3^ mg mL^–1^ for the adapted UV–vis method, and from 7.33 × 10^–4^ to 4.83 × 10^–3^ mg mL^–1^ in the DIA-RD method, in agreement with the limits of ANVISA, FAO, and WHO legislation. Thus, the proposed methods showed great potential for application to the quality control of commercial candies containing E129 dye and meeting the principles of GAC.

## Supplementary Material



## Notes

Graph of the analytical curve of dye E129 and the visualization of the solutions prepared with 5% (*v*/*v*) ammonia methanol solution; normal plot and Pareto diagram of 2^2^ full factorial design for DIA-RD method; response surface plot of 2^2^ complete factorial designs for DIA-RD method; results of the 2^2^ full factorial design, performed in duplicate with five central points, for analysis of the analytical curves by DIA-RD method (−1: minimum intensity; 0: off; +1: maximum intensity); multivariate chemometric model plot by multiple capture and analytical curve prediction model metrics with DIA-RD method; literature studies using DIA methods for the quantification of artificial colors in foods; GAPI parameters for analytical methods used to determine E129 dye in soft candies; and analytical Greenness report sheet AGREE (PDF)
