# Supplementary material for: Development of a Fast, Low-Cost, and Green Method to Quantify Allura Red AC Dye in Candies through Digital Images Using a Smartphone
Source: ACS Omega. 2025 Oct 9;10(48):58514–21. doi: 10.1021/acsomega.5c06279 (PMC12771147; doi:10.1021/acsomega.5c06279)
Supplement: Supplementary file 1 [file ao5c06279_si_001.pdf]

Supplementary material

Development of a fast, low-cost, and green method to quantify Allura Red AC dye in candies through digital images using a smartphone

Maria Eduarda Bezerra Coutinho, Bruna Ramos de Souza Gomes, Jandyson Machado Santos*

Petroleum, Energy and Mass Spectrometry Research Group (PEM), Department of Chemistry, Federal Rural University of Pernambuco (UFRPE), Recife, Pernambuco, 52171-900, Brazil

*Corresponding author: E-mail: jandyson.machado@ufrpe.br (Santos, J.M).

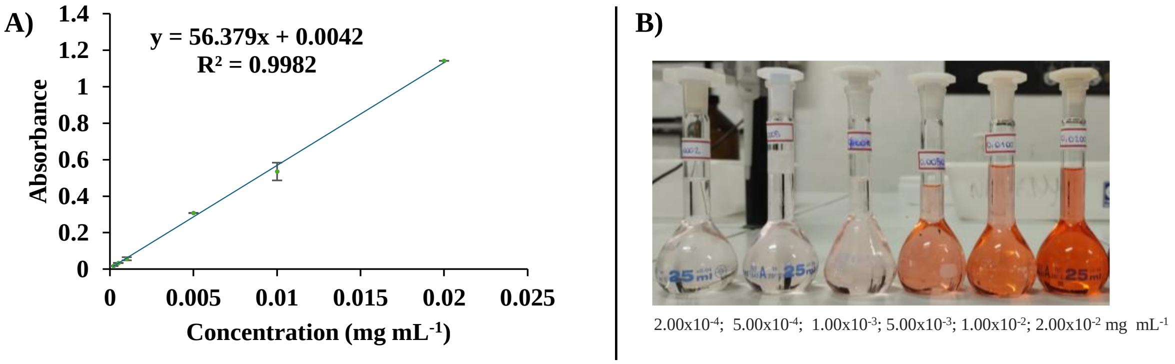

Fig. S1 Graph of the analytical curve of dye E129 (A) and the visualization of the solutions prepared with 5% (*v/v*) ammonia methanol solution (B) as described in the reference method (n=3).

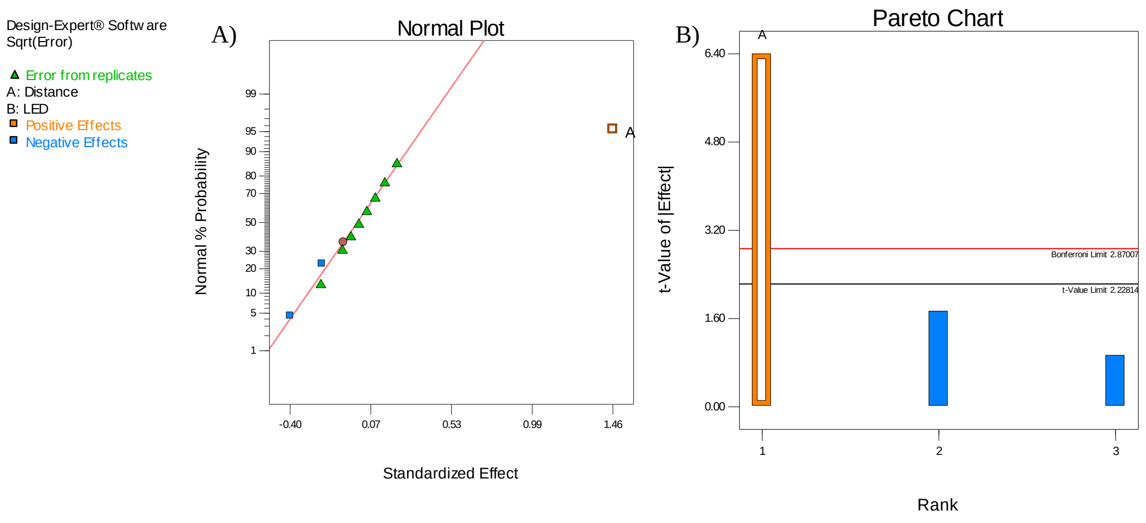

Fig. S2 Normal plot (A) and Pareto diagram (B) of 2^2 full factorial design (A: Distance factor; B: LED Factor) for DIA-RD method.

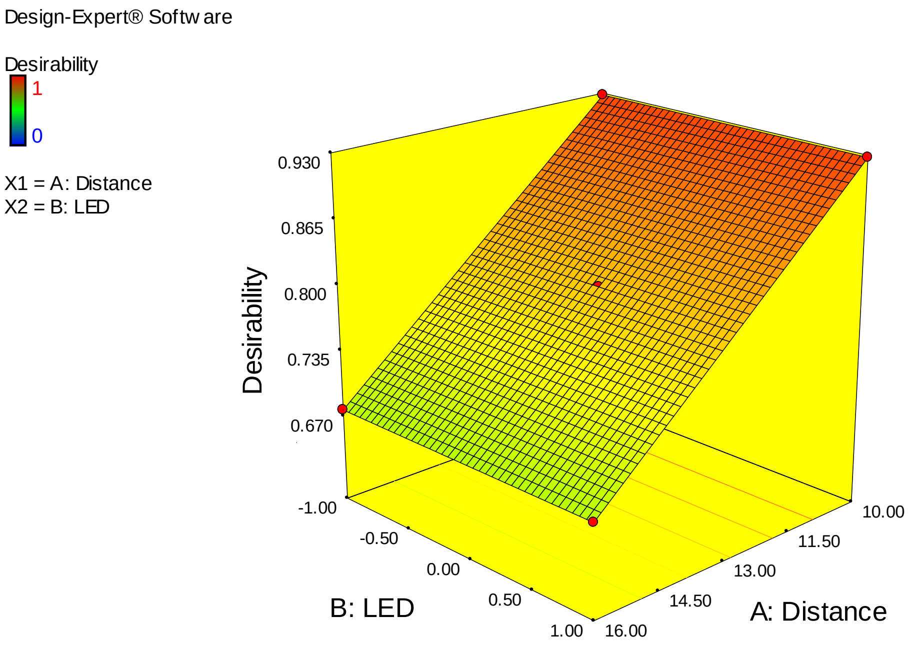

Fig. S3 Response surface plot of 2^2 complete factorial designs for DIA-RD method.

Table S1 Results of the 2^2 full factorial design, performed in duplicate with five central points, for analysis of the analytical curves by DIA-RD method (-1: Minimum intensity; 0: off; +1: Maximum intensity).

Run	Factor Distance (cm)	Factor LED	Relative Error (%)
**T1**	13	0	36.91
**T2**	16	+1	3.24
**T3**	16	-1	7.13
**T4**	10	+1	1.34
**T5**	13	0	38.38
**T6**	13	0	37.77
**T7**	16	+1	5.55
**T8**	10	-1	1.35
**T9**	16	-1	7.31
**T10**	13	0	39.65
**T11**	10	-1	0.76
**T12**	10	+1	0.26
**T13**	13	0	32.25

Table S2 Multivariate chemometric model plot by multiple capture and analytical curve prediction model metrics with DIA-RD method.

Chemometric model – RD	Results
**Correlation coefficient**	0.99
**Relative Error (%)**	4.63×10^{-1}
**RMSEP**	0.00
**RMSEC**	4.75×10^{-5}
**Bias**	5.30×10^{-18}

Table S3 Literature studies using DIA methods for the quantification of artificial colors in foods.

References	Analytes	Samples	Software	Tools	Linear range (mg mL^{-1})	LD and LQ (mg mL^{-1})
Proposed method	E129	Candies	REDGIM	Smartphone	2.00×10^{-4} to 2.00×10^{-2}	LD: 5.51×10^{-5} LQ: 1.67×10^{-4}
1	E132	Candies	ImageJ	HP Photosmart	1.00×10^{-4} to 5.00×10^{-3}	LD: 2.00×10^{-4} LQ: 6.80×10^{-4}
2	E102 and E129	Liquid sweetener, liqueur, popsicles, and liquid candy	Image Processing Toolbox, PLS-Toolboox and MATLAB	Flatbed scanner	–	LD: E102: 1.80×10^{-3} E129: 6.00×10^{-4}
3	E129	Hard candies	PLS Toolbox, Image Processing Toolbox and MATLAB	Smartphone	2.29×10^{-2} to 7.88×10^{-2}	–
4	E110	Beverages	MATLAB, PLS Toolbox, Image Processing Toolbox	Flatbed scanner	7.80×10^{-3} to 3.97×10^{-2}	–
5	E105, E110, E122 and E133	Chocolate, jelly, and gelatin	Color Grab	Smartphone	2.00×10^{-2} to 5.0×10^{-1}	LD: 4.55×10^{-3} to 5.86×10^{-3}
6	E122, E133, E110 and E104	Gelatin, chocolate, and beverages	Microsoft Paint, Photoshop and MATLAB	Flatbed scanner	2.00×10^{-2} to 2.50×10^{-1}	LD: 4.82×10^{-3} to 8.05×10^{-3}

Table S4 GAPI parameters for analytical methods used to determine E129 dye in soft candies.

Method / Category	Reference method	Adapted UV-Vis method	DIA-RD method
**(P1) Sample collection, preservation, transport and storage**			
**(1) Collection**	-	-	-
**(2) Preservation**	None	None	None
**(3) Transport**	None	None	None
**(4) Storage**	Store the sample at room temperature and without light	None	None
**(P2) Sample preparation**			
**(5) Type of method: direct or indirect**	Indirect – Extraction required	Indirect – Filtration required	Indirect – Filtration required
**(6) Scale of sample preparation**	Extraction	-	-
**(7) Solvents/reagents used**	Non-green	Green solvents/reagents used	Green solvents/reagents used
**(8) Additional treatments**	Centrifugation	None	None
**(P3) Reagent and solvents**			
**(9) Amount**	>100 mL	<10 mL	<10 mL
**(10) Health hazard**	Moderately toxic; could cause temporary incapacitation; NFPA = 2 or 3	NFPA health hazard score = 0	NFPA health hazard score = 0
**(11) Safety hazard**	The highest NFPA flammability or instability score of 2 or 3, or a special hazard is used.	The highest NFPA flammability or instability score of 0	The highest NFPA flammability or instability score of 0
**(P4) Instrumentation**			
**(12) Energy**	>1.5 kWh per sample	>1.5 kWh per sample	<0.1 kWh per sample
**(13) Occupational hazard**	Emission of vapors to the atmosphere	None	None
**(14) Waste**	>10 mL	1–10 mL	1–10 mL
**(15) Waste treatment**	Require passivation	-	-
**(P5) Quantification**			
**Central Circle**	Yes	Yes	Yes

Table S5 Analytical Greenness report sheet AGREE (Methods: Reference, Adapted UV-Vis and DIA-RD).

Analytical Greenness report sheet			
**Category** / **Method**	**Reference method**	**Adapted UV-Vis method**	**DIA-RD method**
**(1) Pre-treatment**	0.90	0.90	0.90
**(2) Sample quantity**	0.47	0.57	0.57
**(3) Analysis**	0.33	0.33	0.33
**(4) Sample preparation steps**	0.00	1.00	1.00
**(5) Automation/Miniaturization**	0.00	0.00	0.50
**(6) Derivatization**	1.00	1.00	1.00
**(7) Waste**	0.08	1.00	1.00
**(8) Throughput**	0.45	0.45	0.45
**(9) Energy**	1.00	1.00	1.00
**(10) Reagents**	0.00	1.00	1.00
**(11) Toxic reagents**	0.00	1.00	1.00
**(12) Operator safety**	0.20	1.00	1.00

References

(1) de Sá, I. C.; Feiteira, F. N.; Pacheco, W. F. Quantification of the Food Dye Indigo Carmine in Candies Using Digital Image Analysis in a Polyurethane Foam Support. *Food Anal Methods* 2020, *13* (4), 962–969. https://doi.org/10.1007/S12161-020-01715-5.

(2) Vidal, M.; Garcia-Arrona, R.; Bordagaray, A.; Ostra, M.; Albizu, G. Simultaneous Determination of Color Additives Tartrazine and Allura Red in Food Products by Digital Image Analysis. *Talanta* 2018, *184*, 58–64. https://doi.org/10.1016/J.TALANTA.2018.02.111.

(3) Botelho, B. G.; Dantas, K. C. F.; Sena, M. M. Determination of Allura Red Dye in Hard Candies by Using Digital Images Obtained with a Mobile Phone and N-PLS. *Chemometrics and Intelligent Laboratory Systems* 2017, *167*, 44–49. https://doi.org/10.1016/j.chemolab.2017.05.004.

(4) Botelho, B. G.; De Assis, L. P.; Sena, M. M. Development and Analytical Validation of a Simple Multivariate Calibration Method Using Digital Scanner Images for Sunset Yellow Determination in Soft Beverages. *Food Chem* 2014, *159*, 175–180. https://doi.org/10.1016/J.FOODCHEM.2014.03.048.

(5) Saadati, M. Smartphone-Based Digital Image Analysis for Determination of Some Food Dyes in Commercial Products. *Food Anal Methods* 2021, *14* (11), 2367–2374. https://doi.org/10.1007/S12161-021-02059-4.

(6) Sorouraddin, M. H.; Saadati, M.; Mirabi, F. Simultaneous Determination of Some Common Food Dyes in Commercial Products by Digital Image Analysis. *J Food Drug Anal* 2015, *23* (3), 447–452. https://doi.org/10.1016/J.JFDA.2014.10.007.
